# Biological Treatment of Psoriasis—Data So Far

**DOI:** 10.3390/ph19020340

**Published:** 2026-02-21

**Authors:** Mateusz Matwiejuk, Agnieszka Mikłosz, Hanna Myśliwiec, Adrian Chabowski, Iwona Flisiak

**Affiliations:** 1 Department of Dermatology and Venereology, Medical University of Bialystok, 15-540 Bialystok, Poland; 2 Department of Physiology, Medical University of Bialystok, Bialystok, 15-222 Bialystok, Poland

**Keywords:** psoriasis, alefacept, adalimumab, etanercept, certolizumab, ustekinumab, ixekizumab, brodalumab, secukinumab, bimekizumab, risankizumab, guselkumab, tildrakizumab

## Abstract

Psoriasis is a chronic, inflammatory skin disease occurring worldwide that significantly affects patients’ quality of life. This common skin condition is characterized by abnormal hyperplasia of keratinocytes, which leads to the formation of raised, scaly plaques, typically located on the head, elbows, knees, and lumbar region. Psoriasis usually requires long-term drug therapy, which aims not only to combat skin symptoms but also to improve quality of life. Although topical treatments, systemic treatments (methotrexate, cyclosporine, acitretin), and phototherapy play a role, biologic agents have improved the efficacy of treatment of moderate-to-severe psoriasis. The purpose of this article is to comprehensively review the clinical trial data and evaluate and compare the key features of the currently approved biologic drugs for the treatment of psoriasis.

## 1. Introduction

Psoriasis is a chronic papulosquamous skin disease affecting 125 million people worldwide and posing a substantial burden for individuals and society [1]. Psoriasis occurs equally in men and women, although women often experience an earlier onset compared to men. In addition, people with a family history of psoriasis are more likely to develop the disease earlier. The age of onset usually shows two peaks: in men, these are 30–39 years and 60–69 years. In turn, in women, the peaks of onset of the disease tend to occur about 10 years earlier than in men [2]. Psoriasis is associated with several medical conditions, including obesity [3], cardiovascular disease [4], hyperuricemia [5,6], chronic obstructive pulmonary disease (COPD) [7] and metabolic syndrome [8]. The most common form of psoriasis vulgaris is characterized by raised, red patches on the skin covered with silvery-white scales. They appear mainly on the elbows, knees, scalp, and lower back. Less common forms of psoriasis include guttate psoriasis, inverse psoriasis, erythrodermic psoriasis, and pustular psoriasis. For instance, guttate psoriasis presents with the appearance of small, scaly papules arranged in a centripetal manner, often triggered by a streptococcal throat infection. Inverse psoriasis appears as smooth, red patches without scales and affects skin folds, such as the armpits, groin, and under the breasts. In turn, pustular psoriasis is morphologically distinct and involves pus-filled blisters on the skin. It can be divided into generalized pustular psoriasis or localized, based on the affected anatomical region of the body. Finally, erythrodermic psoriasis is a severe, potentially life-threatening form of psoriasis that covers most of the total body surface area with confluent erythema, scales, or exfoliation [9]. Although psoriasis cannot be cured, treatment should include early identification and the treatment of patients at an early stage of the disease process to minimize the physical and psychological consequences of the disease. In general, when selecting a treatment strategy for plaque psoriasis, it is important to consider the severity of the disease, its location, and the presence of comorbidities, as well as patient preferences and satisfaction. In the 19th century, arsenic was used to treat psoriasis, but its effectiveness was limited, and the risks were significant. High doses of arsenic were associated with ocular and gastrointestinal tract disturbances [10]. Topical therapies have been used to treat psoriasis, including salicylic acid (to soften and remove scales from the skin) and coal tar, which has anti-inflammatory and anti-proliferative properties. In turn, the highly effective Dithranol (anthraline) can cause skin irritation and discolouration. After the discovery of corticosteroids, the use of 17-hydroxycorticosterone-21-acetate (an early topical steroid) was documented in 1952 in two patients with psoriasis. However, the therapeutic effect was virtually nonexistent, which may have been due to the low potency of the specific steroid used at the time [11]. Methotrexate was first used to treat psoriasis in the early 1950s [12]. It is now considered the first-line systemic therapy for many people with psoriasis. Methotrexate is also an effective treatment for psoriatic arthritis [13]. The breakthrough discovery of the effectiveness of combining psoralens (both topical and oral) with UVA light (PUVA therapy) in the treatment of psoriasis occurred in 1973 [14]. PUVA therapy has since become a well-established treatment option for psoriasis, although its use has become more nuanced due to the potential long-term risk of skin cancer, squamous cell carcinoma of the skin [15]. Furthermore, the efficacy of cyclosporine in the treatment of psoriasis was first documented in 1979 in patients with psoriatic arthritis [16]. However, high doses of cyclosporine and long-term use lead to the development of arterial hypertension and show nephrotoxicity [17]. In turn, acitretin was introduced in the late 1980s as an improvement over etretinate due to its more favorable pharmacokinetic profile. The mechanism of action of acitretin is to reduce the excessive growth and proliferation of skin cells observed in psoriasis [18]. Interestingly, the combination of acitretin with PUVA therapy may be more effective than either treatment used alone [19]. The use of biologics has greatly improved the treatment of psoriasis and psoriatic arthritis [20].

Skin inflammation in psoriasis involves a complex interaction between keratinocytes, natural killer T (NKT) cells, plasmacytoid dendritic cells (pDCs), and macrophages. Briefly, activated myeloid dendritic cells produce cytokines like IL-12 and IL-23 that play a crucial role in the immunopathogenesis of psoriasis. Helper T cells type 1 (Th1), type 17 (Th17), and type 22 (Th22) are involved in the inflammatory response. IL-17, produced by Th17 cells, is a key driver of inflammation in psoriasis [1]. In general, the immunomodulatory effect of biological therapies is mediated via their actions against these cytokines (i.e., IL-17 or IL-23) and TNFα. In recent years, biological therapies have revolutionized the treatment of psoriasis. This narrative review presents the efficacy of selected biological therapies in the treatment of psoriasis [20]. The examples and comparisons of biologics are presented in Table 1.

## 2. Materials and Methods

A medical literature search of PubMed (1952–present) and Google Scholar, conducted between September and October 2025, was performed using appropriate terms without date limitations. The main object of the research was to identify the therapeutic effect of biologic treatment in psoriasis. Medical subject headline terms included “alefacept in psoriasis”, “adalimumab in psoriasis”, “etanercept in psoriasis”, “certolizumab in psoriasis”, “ustekinumab in psoriasis”, “ixekizumab in psoriasis”, “brodalumab in psoriasis”, “secukinumab in psoriasis”, “bimekizumab in psoriasis”, “risankizumab in psoriasis”, “guselkumab in psoriasis” and “tildrakizumab in psoriasis”. In total, we included 94 articles in our review.

In this article, in the process of looking at the articles searching, we tried to focus mainly on population/participants, intervention/exposure, comparison/control, outcomes, study design, language, publication date, and setting.

Inclusion criteria for selected publications were focusing on the following: population: adults (≥18 years) and children diagnosed with moderate-to-severe chronic plaque psoriasis; study types: randomized controlled trials (RCTs), cohort studies, systematic reviews/meta-analyses addressing biologic therapies; interventions: biologic agents approved for psoriasis (e.g., TNF inhibitors, IL-17/IL-23 inhibitors); comparators: placebo, conventional systemic therapies, or head-to-head biologic comparisons; outcomes: PASI responses (e.g., PASI 75, PASI 90), safety/tolerability, quality-of-life measures; publication criteria: articles published in peer-reviewed scientific journals, English language (if applicable), published within a defined timeframe (e.g., 1952–2025).

As exclusion criteria, we considered: studies using animal models, editorials, narrative reviews, and non-peer-reviewed sources; studies focusing on conditions other than psoriasis, such as other autoimmune diseases, unless they specify psoriasis sub-analyses; studies without clearly reported outcome metrics relevant to biological treatment efficacy.

Afterwards, the titles and abstracts of the searched studies were independently screened by two reviewers (M.M. and A.M.) in order to identify relevant articles that addressed the review subject. Disagreements between reviewers were resolved by a third reviewer (H.M.). Finally, the selected eligible articles were fully reviewed by the rest of the authors.

## 3. Discussion

### 3.1. Blockage of T Cells

#### Alefacept

Alefacept, a fully human recombinant dimeric fusion protein, was one of the first biological agents approved for psoriasis treatment. The drug induces apoptosis of effector (activated) memory T lymphocytes. [21]. Krueger et al. [22] in a multicenter, randomized, double-blind, parallel-group study showed a substantial improvement in all measures of chronic plaque psoriasis. Twenty-eight percent of patients treated with alefacept achieved at least a 75% reduction in Psoriasis Area and Severity Index (PASI), compared to only 8% in patients receiving a placebo. Patients who responded well to the first course of alefacept therapy maintained significant improvement for an average of over 7 months. After the second course of therapy, the majority of patients achieved at least a 50% reduction in PASI and nearly half of patients achieved a 75% or greater reduction in PASI after two courses. Both one or two courses of alefacept were generally well tolerated [22].

Alefacept was withdrawn from the market in 2011 because newer, more effective, and better tolerated biologic drugs (like TNF inhibitors and IL-12/23 blockers) became available, making it less competitive despite its unique ability to induce remission in some psoriasis patients [23,24].

### 3.2. TNF-Alpha Inhibitors

#### 3.2.1. Adalimumab

Adalimumab is the first fully human IgG1 therapeutic monoclonal antibody thattargets and neutralizes tumor necrosis factor-alpha (TNF-α). Adalimumab binds specifically to both transmembrane and soluble forms of TNF-α, preventing TNF-α from interacting with its receptors, TNF-R1 and TNF-R2. This blocks downstream immune signaling and reduces inflammation [25]. Matucci-Cerinic et al. [26] demonstrated the efficacy of adalimumab in the treatment of various forms of psoriasis, as well as psoriatic arthritis. It is a first-line drug, especially in specific patient groups (enthesitis, peripheral arthritis, axial involvement, IBD, uveitis). However, adalimumab is used after the failure of psoriasis treatment with etanercept [26].

Yuksek et al. [27] observed significant improvement in erectile function in male psoriatic patients after adalimumab treatment. Adalimumab therapy was associated with improved sperm motility, vitality, and testosterone levels. These positive effects are likely attributed to adalimumab’s ability to inhibit TNF-α, a pro-inflammatory cytokine. By reducing systemic inflammation, adalimumab may mitigate the negative impact of psoriasis on the male reproductive system [27].

Alabas et al. [28] compared the efficacy and durability of adalimumab versus methotrexate in the treatment of psoriasis. Patients receiving adalimumab were significantly more likely to achieve clear or almost clear skin (PASI ≤ 2) compared with those receiving methotrexate. Moreover, patients receiving adalimumab were less likely to discontinue treatment compared with those receiving methotrexate. This suggests better tolerability and potentially fewer side effects for adalimumab therapy [28].

Menting et al. [29] highlighted the importance of therapeutic drug monitoring for adalimumab in the treatment of psoriasis. The study established a therapeutic dose of adalimumab from 3.51 mg/L to 7.00 mg/L. Patients who achieved PASI 75 had significantly higher adalimumab concentrations (6.07 mg/L) compared with those who did not reach this level of improvement (2.99 mg/L). Notably, one-third of patients in the study had adalimumab concentrations above the upper limit of the therapeutic range, suggesting potential overtreatment. The study used receiver-operator characteristic (ROC) analysis to determine the lower limit of the therapeutic range, yielding an area under the curve (AUC) of 0.756, indicating good diagnostic accuracy. The concentration–effect curve was used to determine the upper limit of the therapeutic range [29].

#### 3.2.2. Etanercept

Etanercept is a biologic TNF-α inhibitor. It is a recombinant fusion protein consisting of two extracellular domains of the human TNF receptor p75 (TNFR2) linked to the Fc portion of human IgG1. Etanercept acts as a soluble receptor that binds and neutralizes both TNF-α and TNF-β (lymphotoxin-α), thereby reducing inflammatory signaling in autoimmune diseases such as psoriasis [30].

Narbutt et al. [31] proved that etanercept is a promising treatment option for pediatric psoriasis. A high percentage of patients achieved substantial improvements in psoriasis symptoms within 16 weeks of treatment with etanercept: the most participants achieved PASI 50, a number of participants achieved PASI 75, and the lowest number of participants achieved PASI 90. The mean body surface area (BSA) affected by psoriasis decreased significantly. In addition, the mean score of the Children’s Dermatology Life Quality Index (CDLQI) score, which measures the impact of psoriasis on quality of life, also decreased substantially. To sum up, the study indicated that etanercept was generally well tolerated by the pediatric population [31].

Lin et al. [32] described a case of a 2-year-old patient with pustular psoriasis who showed significant improvement after changing the treatment from cyclosporine to etanercept. The patient was initially treated with cyclosporine, but this only partially suppressed the expression of neutrophil activation-related genes involved in the inflammation process. Therefore, the patient received an etanercept, an anti-TNF-α inhibitor, at a dose of 25 mg divided over three weeks (0.98 mg/kg/week). Within 2 weeks of treatment with etanercept, almost complete remission of skin lesions (especially pustules and erythema) was observed, and the white blood cell count decreased to 1.69 × 10^10^/L. RNA sequencing analysis of peripheral blood mononuclear cells revealed that etanercept effectively downregulated the expression of genes related to neutrophil activation, neutrophil-mediated immunity, and degranulation compared with cyclosporine. This suggests that etanercept may have a more potent effect on the underlying inflammatory processes driving the skin condition [32].

Huynh et al. [33] suggested considering the phenomenon of “paradoxical colitis” in patients treated with biologics like etanercept and other IL-17 inhibitors. A 46-year-old woman with psoriasis and psoriatic arthritis experienced gastrointestinal symptoms after treatment with etanercept. She was subsequently treated with other biologics (certolizumab, tofacitinib), potentially masking the underlying cause of her gastrointestinal issues. She was diagnosed with paradoxical colitis, probably caused by etanercept. Discontinuation of etanercept resulted in improvements of gastrointestinal symptoms [33].

#### 3.2.3. Certolizumab

Certolizumab pegol (CZP) is a PEGylated anti-TNF-α biologic. It consists of a humanized Fab′ fragment of a monoclonal antibody directed against TNF-α, covalently linked to polyethene glycol (PEG). The PEGylation prolongs the molecule’s half-life and enhances its stability, thereby increasing circulation time and therapeutic potential. Unlike most other anti-TNF-α biologics, CZP lacks the Fc portion of the antibody. This structural difference alters its interaction with the immune system and significantly reduces placental transfer during pregnancy. By binding to both soluble and membrane-bound TNF-α, certolizumab pegol inhibits its interaction with TNF-α receptors (TNFR1 and TNFR2), thereby decreasing inflammation by blocking a key pro-inflammatory cytokine involved in autoimmune diseases such as psoriasis [34].

Korge et al. [35] investigated the efficacy and safety of certolizumab pegol (CZP) in the treatment of moderate-to-severe psoriasis. CZP significantly improved psoriasis symptoms. Precisely, a high PASI 75 in the majority of participants and PASI 90 in nearly half of patients response rates were observed after 12 months of therapy, indicating significant skin clearance. Patients with lower baseline PASI scores showed even greater improvement over time. Moreover, DLQI scores decreased from 12.4 to 2.3, indicating a substantial improvement in the patients’ quality of life. A larger percentage of patients achieved minimal or no impact of psoriasis on their daily lives (DLQI 0/1) after 12 months. The high rate of persistence over the last 1 year of CZP treatment was about 85%, suggesting good tolerability. More than thirty percent of patients did not experience any adverse events. Approximately 9.3% of adverse events were serious, with infections and infestations being the most common. Importantly, serious adverse events related to CZP treatment were rare [35].

Ruiz et al. [36] investigated the efficacy of CZP in patients with moderate-to-severe plaque psoriasis. The authors observed a high treatment efficacy, with 90.9% of patients achieving the predefined therapeutic target. Significant improvements were observed in PASI, BSA, Physician’s Global Assessment (PGA), Nail Psoriasis Severity Index (NAPSI), and DLQI. The mean absolute PASI score decreased by 8 points indicating a substantial improvement in psoriasis severity. Reductions were also observed in other measures of psoriasis severity, including BSA (by 11.3), PGA (by 1.9), and NAPSI (by 3.3). The mean DLQI score decreased by 9.0 points, suggesting a significant improvement in the patient’s quality of life. Generally, CZP was reported to be well tolerated. Only 3% of patients discontinued treatment, although 9% did not reach the initial treatment goal. In summary, CZP is a highly effective treatment option for moderate-to-severe plaque psoriasis [36].

Coto-Segura et al. [37] presented a sophisticated computational approach to modeling the effects of CZP in patients with moderate-to-severe psoriasis. The researchers used a “virtual population” (vPop) approach, creating a computer-simulated population of patients that reflected the characteristics of psoriasis patients with comorbidities and disease severity. Physiologically based pharmacokinetic (PBPK) models were used to simulate how CZP would be absorbed, distributed, metabolized, and eliminated in each virtual patient. Systems Biology (SB) models were incorporated to simulate the biological mechanisms of psoriasis and the interactions of CZP with these pathways. The vPop was simulated using two validated CZP dosing regimens: 200 mg every two weeks and 400 mg every two weeks. Both regimens included a loading dose of 400 mg at weeks 0, 2, and 4. The model predicted clinical outcomes (like PASI scores) and molecular markers of psoriasis severity. The model identified clusters of virtual patients based on their predicted protein activity responses to CZP, potentially revealing subgroups that may respond differently to treatment. To sum up, this study demonstrates the power of in silico modeling in drug development and personalized medicine [37].

### 3.3. Blockage of IL-12/IL-23

#### Ustekinumab

Ustekinumab is a fully human IgG1κ monoclonal antibody targetting the shared p40 subunit of IL-12 and IL-23. By binding to this subunit, it prevents these cytokines from interacting with the IL-12Rβ1 receptor on immune cells. As a result, it significantly reduces the activity of the Th1 and Th17 inflammatory pathways, which play a central role in the pathogenesis of diseases such as psoriasis [38].

Feldman et al. [39] found that SB17, a biosimilar to ustekinumab, was clinically comparable to ustekinumab in the 28 weeks of study in patients with moderate-to-severe plaque psoriasis. The adjusted difference in change from baseline in PASI at week 12 was −0.6% (95% CI: −3.780, 2.579), within the noninferiority margin, indicating comparable efficacy of SB17 and UST. In addition, the PGA and DLQI showed similar results for both treatments [39].

Li et al. [40] examined the factors influencing the short-term efficacy of ustekinumab. The average PASI score, a measure of psoriasis severity, decreased significantly from 9.4 at baseline to 3.7 at week 4 after the first dose of ustekinumab. More than thirty percent of patients achieved a PASI 75 score after the first dose. About nineteen percent of patients achieved a PASI 90 score, whereas one-fifth of patients achieved complete clearance of psoriasis. Patients without metabolic syndrome had significantly higher rates of achieving PASI 75, and, furthermore, lower triglyceride levels were associated with a greater likelihood of achieving PASI 75. Female patients, as well as patients with a family history of psoriasis, had a higher rate of achieving PASI 100. On the other side, the average BSA affected by psoriasis also decreased significantly from 11.2% to 4.8% within the same timeframe. Higher levels of HDL-C were associated with increased odds of achieving all three treatment goals (PASI 75, 90, and 100). Importantly, some factors like a healthier metabolic profile (lower triglycerides, higher HDL-C), female gender, and a family history of psoriasis may increase the chances of a better response to this medication [40].

Gonulai et al. [41] reported the efficacy of ustekinumab in patients who had previously tried other treatment options (non-naïve patients). A high percentage (80.55%) of non-naïve patients responded positively to ustekinumab treatment. A large proportion of patients achieved a substantial improvement in their psoriasis symptoms (PASI 75). Male patients generally experienced a faster and robust response to ustekinumab compared to female patients. However, the study did not find any significant correlation between the response to treatment (PASI 75, 90, 100) in naïve and non-naïve patients [41].

Kong et al. [42] presented a case of a 71-year-old man with psoriasis who developed bullous pemphigoid (BP) twice after receiving ustekinumab injections. The patient experienced the onset of blisters on his upper limbs shortly after the first and second ustekinumab injections. The patient had elevated IgE levels and eosinophil count. Enzyme-linked immunosorbent assay showed positive serum IgG antibodies against BP180 and BP230. Histological findings were consistent with BP, subepidermal blister and superficial dermal inflammation comprising lymphocytes and eosinophils. Direct immunofluorescence showed IgG deposits along the basement membrane zone. Discontinuation of ustekinumab and initiation of treatment with methotrexate, minocycline, and topical corticosteroids brought both psoriasis and BP under control [42].

Olteanu et al. [43] presented a case of a 39-year-old woman with Crohn’s disease who developed a psoriasiform rash while receiving ustekinumab. She was subsequently treated with adalimumab, which controlled her Crohn’s disease and resolved the psoriatic rash. The patient experienced flares of Crohn’s disease while receiving adalimumab. She was treated again with ustekinumab, which resulted in clinical remission of her Crohn’s disease for three years. Psoriatic lesions developed while receiving ustekinumab. Skin biopsy confirmed typical features of psoriasis: epidermal hyperplasia, parakeratosis, Munro’s microabscesses, thinned granular cell layer and dilated dermal capillaries. Finally, topical beclomethasone ointment was added to ustekinumab therapy, which resulted in the resolution of psoriatic lesions [43].

### 3.4. Blockage of IL-17

#### 3.4.1. Ixekizumab

Ixekizumab is a humanized IgG4 monoclonal antibody that specifically targets and blocks the pro-inflammatory cytokine IL-17A, effectively treating moderate-to-severe plaque psoriasis and psoriatic arthritis by stopping the inflammatory cascade driven by IL-17A binding to its receptor, leading to significant skin clearing [44].

Valenti et al. [45] demonstrated the efficacy of ixekizumab in the treatment of plaque psoriasis, focusing particularly on difficult areas such as the scalp, palms, soles, nails, and genitals. High efficacy was observed: for the scalp, 96% of patients achieved clear or almost clear skin at one year; for the palmoplantar area, 95.6% of patients experienced significant improvement; for the genitals, 95.2% of patients experienced substantial clearance; for nails, 85% of patients achieved clear or almost clear nails. Importantly, no serious adverse events were reported. In conclusion, ixekizumab may be an effective treatment option for plaque psoriasis, particularly in difficult-to-treat areas like the scalp, palms, soles, nails, and genitals [45].

Similarly, Burlando et al. [46] found that ixekizumab is an effective and well tolerated treatment for moderate-to-severe plaque psoriasis. Ixekizumab not only achieved significant improvement in PASI 75 responses but also in PASI 90 and 100 responses. These improvements were sustained in most patients for up to three years. Moreover, the study did not find a significant difference in efficacy between patients previously treated with biologics (bio-switch) and those who were not (bio-naive). Among the side effects, two cases of eczema were observed, which led to the discontinuation of treatment. Ixekizumab was generally well tolerated, and no serious adverse events were reported [46].

Deng et al. [47] investigated the metabolic changes associated with two different psoriasis treatments: adalimumab and ixekizumab. The researchers spotted few changes in lipids: Cer-NS (d18:2/24:0), Cer-NS (d18:1/24:1) were upregulated; FAHFA (18:0/20:2), glycerol 1-hexadecanoate, and arachidonic acid were downregulated. Among glycerophospholipids, phosphatidylcholine was upregulated, whereas phosphatidylethanolamine was downregulated; for lysophosphatidylcholine and lysophosphatidylethanolamine levels, saturated forms were downregulated, while unsaturated forms were upregulated; similarly, unsaturated forms were upregulated in lysophosphatidic acid. Furthermore, pathway enrichment analysis revealed significant changes in phenylalanine metabolism and arachidonic acid metabolism, particularly with ixekizumab treatment. Ixekizumab appears to have a more substantial impact on the metabolome compared to adalimumab in patients with psoriasis. In general, these metabolic changes involve different lipid classes, suggesting a complex interplay of lipid signaling pathways in response to these treatments. It seems that biologics, particularly ixekizumab, may affect glycerophospholipid metabolism and potentially contribute to a shift from a pro-inflammatory to an anti-inflammatory metabolic state in patients with psoriasis [47].

Tamer et al. [48] examined the impact of different biologics on the monocyte-to-high-density lipoprotein cholesterol ratio (MHR) in patients with psoriasis. The authors found that MHR decreased significantly after treatment with ixekizumab. However, no significant differences in MHR were observed in psoriatic arthritis or other comorbidities. Males had significantly higher baseline MHRs compared to females. Since a high MHR is associated with increased cardiovascular risk, the reduction in MHRs observed with ixekizumab may suggest a potential cardiovascular benefit of this treatment. In summary, MHR could be a useful biomarker for selecting appropriate biologic therapy and monitoring treatment response [48].

Ozdemier et al. [49] reported successful treatment with ixekizumab in a difficult case of generalized pustular psoriasis of pregnancy (GPPP) in a 31-year-old woman. The patient had a history of GPPP during previous pregnancies, emphasizing the recurrent nature of the condition in some individuals. First, infliximab initially controlled the condition, but the patient discontinued it. Second, adalimumab maintained a 2-year remission but was discontinued before the current pregnancy. Third, certolizumab showed an initial partial response but later exacerbated the condition. Infliximab then provided some control of the condition during pregnancy, but exacerbations occurred during steroid tapering. In the case of a postpartum exacerbation, a severe postpartum exacerbation occurred despite continued infliximab therapy. Finally, switching to ixekizumab postpartum led to the complete resolution of pustules and erythema within 2 weeks, with sustained remission for 1 year [49].

Gottlieb et al. [50] compared the effectiveness of ixekizumab and etanercept in the treatment of psoriasis. Unlike etanercept, ixekizumab showed similar high efficacy in patients who had previously use biologics (biologic-experienced) and those who hadn’t (biologic-naive). At all measured PASI scores (PASI 75, PASI 90, and PASI 100), ixekizumab was significantly more effective than etanercept, regardless of prior biological usage. The study examined ixekizumab administered every 2 weeks and every 4 weeks. Both were superior to etanercept, but ixekizumab administered every 2 weeks generally showed the highest response rates [50].

Bucur et al. [51] reported that ixekizumab offered better long-term efficacy and tolerability than secukinumab in the treatment of psoriasis. Ixekizumab was used for a longer period of time compared with secukinumab, suggesting better long-term efficacy and tolerability of the latter. Furthermore, fewer patients treated with ixekizumab changed therapy because of adverse events or ineffectiveness compared with those treated with secukinumab. Interestingly, in those switching from ixekizumab, the change in drug occurred later than in those switching from secukinumab. Kaplan–Meier curves illustrated the proportion of patients remaining on each treatment over time. The data showed a clear separation, with ixekizumab consistently showing higher retention rates at both 2 and 4 years. For example, after 2 years, 88% of patients who used ixekizumab were still on treatment compared with 75% of patients treated with secukinumab. The median duration of treatment for patients on ixekizumab was 43 months compared with 37 months for secukinumab. Younger patients (under 50) tended to have longer drug survival on ixekizumab compared to older patients. This difference appeared relatively early in the treatment (approximately 10 months). Of the other comorbidities, only psychological impairment was statistically associated with switching in the secucinumab arm (*p* = 0.046). No such association was found in the ixekizumab arm [51].

Ting et al. [52] compared the efficacy and drug survival of several biologic treatments for psoriasis. Ixekizumab had a good 1-year survival rate (87.2%) but a lower 5-year survival rate (59.4%). Both ixekizumab and guselkumab demonstrated the achievement of higher PASI 75 response rates (94.9% and 93.8%, respectively), representing a 75% improvement in psoriasis severity. The median time to achieve PASI 75 varied across treatments; ixekizumab had the highest rate of PASI 90 achievement (84.7%), followed by guselkumab (78.1%). Similarly, ixekizumab had the highest PASI 100 achievement rate (71.2%). The most common reasons for discontinuation were a lack of initial efficacy and loss of efficacy over time [52].

#### 3.4.2. Secukinumab

Secukinumab is a fully human monoclonal IgG1 antibody that selectively binds to the pro-inflammatory cytokine interleukin-17A (IL-17A), preventing it from interacting with its receptor and thereby reducing inflammation in psoriasis [53].

Papp et al. [54] reported an analysis of the PURE study, focusing on the effectiveness and safety of secukinumab (IL-17A inhibitor) in patients with plaque psoriasis, comparing standard dosing with dose escalation. A significant reduction in PASI was observed, from a baseline of 13.6 to 1.2 at 36 months. The treatment persistence was high (73% at 40 months). In addition, over seventy-three percent of patients achieved clear or almost clear skin as measured by the Investigator’s Global Assessment (IGA) 0/1 after 36 months. Patients were then administered one of the following secukinumab regimens: 300 mg every 2 weeks, 300 mg every 3 weeks, 450 mg every 4 weeks, or 450 mg every 3 weeks. Almost fifty-eight percent of patients showed improvements in the PASI score after updosing of secukinumab. Treatment persistence was 50% at 12 months after updosing and 40% achieved IGA 0/1 at 15 months. Briefly, the study showed that while the standard dose of secukinumab was highly effective and well tolerated, a subset of patients required dose escalation. Interestingly, while updosing did lead to improvement in some psoriatic patients, the overall response and persistence were lower than in those maintained on the standard dose. Finally, the safety profile remained consistent even if higher doses were implemented [54].

Dauden et al. [55] presented a dose reduction strategy (SEC-DR) in a retrospective, multicenter cohort study of patients with plaque psoriasis who had achieved a sustained response to a standard dose of secukinumab. The dose reduction strategy involved either increasing the interval between doses (51 patients) or reducing the monthly dose to 150 mg (12 patients). SEC-DR was successful in four-fifths of the patients; they maintained PASI responses until the end of the study. This study suggests that a dose reduction strategy for secukinumab may be a viable option for some patients with sustained response to standard therapy [55].

To identify polymorphisms associated with secukinumab response in psoriasis patients in a daily practice setting, Munoz-Aceituno et al. [56] identified single-nucleotide polymorphisms (SNPs) associated with secukinumab response in patients with plaque psoriasis. At 6 months, 67% of psoriatic patients achieved PASI ≤ 3, and 65% of patients with psoriasis achieved PASI ≤ 1. Subsequently, at 12 months, 75% achieved PASI ≤ 3, and 64% achieved PASI ≤ 1. Multivariable analysis identified four SNPs as risk factors (associated with not achieving PASI ≤ 3): rs1801274 (FCGR2A), rs2431697 (miR-146a), rs10484554 (HLCw6) and one protective factor (associated with achieving PASI ≤ 3), rs1051738 (PDE4A). At 12 months, in patients with PASI ≤ 3, univariate analysis revealed several SNPs associated with PASI response. A rs26528 (IL27), genotype CC, was protective (associated with achieving PASI ≤ 3), whereas, rs12191877 (HLACw6), genotype TT, increased the risk of not achieving PASI ≤ 3 (non-responder). A number of polymorphisms associated with response to secukinumab have been identified as capable of predicting the potential response or lack of response to this drug in patients with plaque psoriasis [56].

Du et al. [57] studied the impact of secukinumab treatment on the gut microbiota of patients with psoriasis and its potential role in predicting treatment efficacy and risks. The authors found significant changes in the gut microbiota of patients after secukinumab treatment compared to both those before treatment and healthy controls. Specific microbiota changes revealed an increased *Firmicutes phylum*, *Ruminococcaceae* family and decreased *Bacteroidota phylum* (leading to an increased *Firmicutes/Bacteroidota* (F/B) ratio). Compared to untreated psoriasis, successful secukinumab therapy was associated with an increased *Firmicutes phylum*, *Pantoea*, and unclassified *Comamonadaceae* genera, and decreased *Bacteroidota phylum*, *Bradyrhizobium*, *Hydrogenophaga*, and *Lactococcus* genera. Secukinumab treatment appeared to promote a more stable gut microbiome homeostasis with functional changes. Moreover, metagenomic analysis revealed alterations in metabolic functional pathways after secukinumab therapy, including downregulation of cardiovascular disease pathways and upregulation of infectious disease pathways. Briefly, secukinumab treatment significantly alters the gut microbiota composition and function in psoriasis patients [57].

Tada et al. [58] in a multicenter, non-interventional, retrospective chart review study evaluated the retention rate and effectiveness of secukinumab in Japanese patients with psoriasis over 5 years. At baseline, the mean PASI score was 9.21 ± 7.37, whereas at week 52, it was 1.4 ± 2.6. However, the most common reason for discontinuation was “insufficient response”. To sum up, this real-world study demonstrated a decent retention rate of secukinumab at 1 year in Japanese psoriasis patients, with significant improvement in PASI scores. Previous use of biologics was identified as a risk factor for treatment discontinuation, and inadequate response was the most common reason for treatment discontinuation [58].

Ma et al. [59] described a potential adverse reaction to secukinumab. An 87-year-old adult patient with ankylosing spondylitis who had been treated with secukinumab for 1 year developed an eczematous rash. The patient was subsequently treated with cetirizine (an antihistamine) and topical hydrocortisone butyrate (a corticosteroid). The skin lesions improved significantly. Secukinumab was discontinued and replaced with adalimumab. The eczematous rash did not recur [59].

Choi et al. [60] reported a case of a 64-year-old female patient who developed a new onset of alopecia areata (AA) after starting secukinumab for psoriasis. Adalimumab was discontinued because of the development of generalized pustular psoriasis, and she was switched to secukinumab for psoriasis. Although her psoriasis symptoms initially improved, six weeks after starting secukinumab treatment, the patient experienced significant hair loss (AA) on her scalp, and her psoriasis worsened. Other causes of hair loss (normal thyroid and ferritin levels) were ruled out. Secukinumab was then discontinued after 2 months, and the patient was switched to guselkumab. Shortly after discontinuing secukinumab and starting guselkumab, her hair began to regrow. It continued to improve, returning to its original density within eight months. Her psoriasis also responded well to guselkumab, although some residual lesions persisted on her hands and feet [60].

Zundell et al. [61] reported a case of an eczema-like reaction potentially related to secukinumab, an IL-17A inhibitor, in a patient with plaque psoriasis. A 45-year-old female with plaque psoriasis, who had been taking secukinumab for four months, presented with a new, intensely itchy rash on her forearms, trunk, and legs. The rash consisted of red, crusted bumps coalescing into eczematous plaques, particularly severe on the palms and soles (volar surfaces). The patient was treated with dupilumab (a monoclonal antibody targeting IL-4 and IL-13). Five months after the dupilumab loading dose, the patient’s original psoriasis recurred, covering 15% of her BSA. The patient then refused further treatment and did not return for follow-up examinations [61].

#### 3.4.3. Bimekizumab

Bimekizumab is a powerful humanized antibody that blocks both pro-inflammatory cytokines IL-17A and IL-17F, offering superior inflammation reduction in psoriasis compared to treatments targeting only IL-17A. By preventing these cytokines from activating the IL-17 receptor, it achieves deeper skin clearance, faster responses, and sustained efficacy, making it a significant advancement in treating psoriasis, with similar safety to other anti-IL-17 drugs [62].

Abdin et al. [63] presented a case report of a 38-year-old male with a 2-year history of untreated plaque psoriasis who developed moderate itching that interfered with his daily life. The patient had 20% of body surface area affected on his trunk, legs, arms, and forehead. He was started on bimekizumab, a dual inhibitor of IL-17A and IL-17F. The patient received a loading dose of 320 mg (two 160 mg subcutaneous injections) and experienced significant itching relief within 6 to 12 h of the injection. There was significant resolution of psoriasis (PASI 90) within 72 h, and complete resolution (PASI 100) within 1 week. The itch score also improved to 0, with only post-inflammatory hyperpigmentation remaining [63].

Rompoti et al. [64] presented the efficacy and safety of bimekizumab, an IL-17A/F inhibitor, in the treatment of plaque psoriasis. Bimekizumab showed high effectiveness in the treatment of plaque psoriasis. At week 4, 65.7% of patients achieved PASI 75, 45.7% achieved PASI 90, and 32.4% people achieved PASI 100. At week 16, response rates increased substantially, with 92.3% of participants achieving PASI 75, 76.9% of patients achieving PASI 90, and 66.7% of examined people achieving PASI 100. Almost ten percent of patients experienced adverse events during the first 24 weeks of treatment; the most common were oral candidiasis (four cases) and bacterial skin infections (two cases). Bimekizumab is a highly effective treatment option for plaque psoriasis, with significant improvement observed as early as week 4 and further increased response rates by week 16 [64].

Similarly, Megna et al. [65] demonstrated that bimekizumab is a promising biologic agent for the treatment of psoriasis. A significant percentage of patients achieved PASI 75, PASI 90, and PASI 100 responses at both weeks 4 and 16. The study compared patients who had never received biologic psoriasis treatment (biologic-naive) with those who had previously received such treatment (biologic-experienced). Interestingly, at baseline, the biologic-naive group had significantly higher PASI and DLQI scores (more severe disease and worse quality of life) than the biologic-experienced group. While not statistically significant, at week 4, a slightly higher percentage of biologic-naive patients achieved PASI 75/90/100 responses compared to biologic-experienced patients. However, by week 16, response rates were similar in both groups, suggesting that bimekizumab is effective even in patients who have previously failed other biologic therapies. Bimekizumab was generally well tolerated. Some adverse events were reported, including candidiasis and eczematous reactions. A small number of patients discontinued treatment because of treatment failure or adverse events. Therefore, bimekizumab is an effective and safe treatment for psoriasis, even in patients who have previously failed other biologic therapies [65].

Hagino et al. [66] performed a real-world study on the effectiveness and safety of bimekizumab for Japanese patients with psoriasis and psoriatic arthritis. The researchers found a rapid and significant decrease in the PASI score by a median of 79.8% score at week 4. This improvement continued gradually thereafter. Improvements were also seen in PASI scores specifically for the trunk, upper, and lower limbs. The authors reported high achievement rates for absolute PASI scores of ≤1 and ≤2, indicating significant disease clearance. For example, at week 16, over 96% of patients achieved a PASI ≤ 2, and over 82% of psoriatic patients achieved a PASI ≤ 1. The authors also assessed PASI 75, 90, and 100, with increasing percentages over time, reaching 75.8%, 62.9%, and 57.6%, respectively, at week 16. Additionally, the neutrophil-to-lymphocyte ratio and neutrophil amount decreased significantly at week 16 compared to baseline, suggesting a potential mechanism of action for bimekizumab. A key finding was that a younger age was associated with a greater percentage reduction in PASI at weeks 4 and 8. This suggests that younger patients may have a better initial response to bimekizumab. Treatment was well tolerated, with no serious or fatal adverse events reported [66].

Blauvelt et al. [67] focused on suicidal ideation and behavior (SIB) and depression in patients with psoriasis. The long-term rate of SIB for bimekizumab therapy was very low (0.13/100 patient-years). This rate falls within the reported ranges for both the general psoriasis population (0.09 to 0.54/100PY) and for patients treated with other biologics like anti-IL-17A/anti-IL-23 therapies (0.09 to 0.19/100PY). This suggests that bimekizumab does not increase the risk of SIB to a greater extent than expected in this patient population. Moreover, screening and monitoring questionnaires also indicated low rates of SIB and depression among patients receiving bimekizumab. At week 16, a higher percentage of bimekizumab-treated patients reported no or minimal depression compared with placebo-treated patients. This suggests a potential benefit of bimekizumab in not aggravating depressive symptoms. Finally, the study also found low scores on standardized scales for suicide risk (Columbia-Suicide Severity Rating Scale) and depression (Patient Health Questionnaire-9) in bimekizumab-treated patients [67].

Reich et al. [68] compared the efficacy and safety of bimekizumab, ustekinumab, and placebo in moderate-to-severe plaque psoriasis. The study enrolled 567 patients who were randomly assigned to one of three groups: bimekizumab (320 mg every 4 weeks), ustekinumab (45 mg or 90 mg every 12 weeks), or placebo. At week 16, bimekizumab had significantly greater efficacy than both ustekinumab and placebo. Firstly, 85% of patients in the bimekizumab group achieved a PASI 90 compared with PASI 50 in the ustekinumab group and 5% in the placebo group. Secondly, 84% of patients in the bimekizumab group achieved an IGA response compared with 53% of patients in the ustekinumab group and 5% in the placebo group. Of note, the risk difference was statistically significant (*p* < 0.0001). Through 52 weeks, treatment-emergent serious adverse events were reported in 6% of patients in the bimekizumab group (including those who switched from placebo at week 16) and 8% of patients in the ustekinumab group. The study showed that bimekizumab is more effective than ustekinumab for treating moderate-to-severe plaque psoriasis [68].

Gordon et al. [69] presented a long-term (56-week) study evaluating the efficacy, duration and safety profile of bimekizumab in the treatment of moderate-to-severe plaque psoriasis. Similar to the above-mentioned studies, bimekizumab was found to be highly effective at week 16. Ninety-one percent of patients receiving bimekizumab achieved PASI 90, compared with only 1% in the placebo group. Furthermore, above ninety percent of patients in the bimekizumab group achieved an IGA score of 0 or 1 (clear or almost clear skin) versus 1% in the placebo group. Importantly, the study showed that these high response rates were maintained through week 56 with both the every 4-week and every 8-week bimekizumab dosing regimens. Bimekizumab was well tolerated, with no unexpected safety findings. During the initial 16-week period, a higher proportion of patients in the bimekizumab group (61%) experienced treatment-emergent adverse events compared with the placebo group (41%). However, from week 16 to week 56, the rates of adverse events were similar across all groups, including the group that switched from placebo to bimekizumab [69].

### 3.5. Blockage of IL-17RA

#### Brodalumab

Brodalumab is a fully human IgG2 monoclonal antibody that specifically targets the interleukin-17 receptor A (IL-17RA). By binding to IL-17RA, it blocks signaling mediated by several cytokines, including IL-17A, IL-17F, the IL-17A/F heterodimer, IL-17C, and IL-25, effectively reducing inflammation, especially in moderate-to-severe plaque psoriasis. Unlike some other IL-17 blockers, it targets the common receptor subunit, providing broad inhibition of IL-17 family signaling, making it a significant treatment option for severe cases [70].

Von Kiedrowski et al. [71] examined the real-world effectiveness and safety of brodalumab for psoriasis treatment in Germany. The study supports the use of brodalumab in routine clinical practice, confirming its previously established efficacy and safety profile. The study included patients who had not previously taken biologics, as well as patients who had, many of whom had previously taken adalimumab or secukinumab. Seventy-four percent of patients achieved a PASI score of 3 or less by week 12, indicating significant improvement in psoriasis severity. The mean PASI score decreased significantly from 17.2 at baseline to 3.3 by week 12. This demonstrates a rapid and significant effect of brodalumab. At approximately 52 weeks, 85.5% of patients achieved a PGA response of 0 or 1 (clear or almost clear skin), and 54.1% were completely clear (PGA 0), which demonstrates the sustained benefit of brodalumab over a year. The efficacy of brodalumab was consistent across patient subgroups, including those with prior biologic experience. The most commonly reported adverse events were nasopharyngitis, psoriasis (paradoxical psoriasis can occur with IL-17 inhibitors), and arthralgia. No new safety concerns were identified [71].

Papp et al. [72] evaluated the effectiveness of brodalumab in psoriasis patients who had an inadequate response to another biologic therapy. Patients with moderate-to-severe psoriasis who had not responded adequately to at least 12 weeks of treatment with a stable dose of another biologic agent were switched to brodalumab (210 mg every 2 weeks) without a washout period. They were followed for 26 weeks. The primary objective was to determine the percentage of patients who achieved complete skin clearance (PASI 100) at week 26. Approximately forty percent of patients achieved PASI 100 at week 16, and this increased to 45.5% at week 26. The average time to achieve PASI 100 was about 97 days, with the median time being 112 days. A total of 53.7% of patients achieved PASI 90 at week 16, increasing to 57.9% at week 26. Adverse events occurred in 64.9% of patients, but most were mild to moderate. Serious adverse events occurred in 4.4% of patients. One case of severe pancreatitis was probably related to brodalumab, leading to the discontinuation of treatment. There were no suicides or deaths. Briefly, this study suggests that brodalumab may be an effective treatment option for patients with psoriasis who have not responded adequately to other biologic therapies. It has shown significant improvements in skin clearance, with a substantial proportion of patients achieving PASI 90 and PASI 100. Although adverse events were common, most were not serious [72].

Gkalpakiotis et al. [73] presented a study of the effectiveness of brodalumab in the treatment of psoriasis. PASI score decreased significantly from a mean of 16.1 at the baseline to 0.5 at the end of the study. In addition, PASI 75 was achieved by 98% of psoriatic patients, PASI 90 by 87.8% of patients suffering from it, and PASI 100 by 75.5% of these patients at approximately 52 weeks. The sPGA scores also improved dramatically, from an average of 3.6 at baseline to 0.4 at the end of therapy. Ninety-two percent of patients achieved an sPGA score of 0 or 1 (clear or almost clear) at week 52. Additionally, the average BSA affected by psoriasis decreased from 20.8% to 0.7%. In addition, DLQI scores, which measure the impact of psoriasis on quality of life, decreased from 14.5 to 1.1. A significant portion of patients (75.6%) reported no impact on their quality of life (DLQI 0 or 1) by the end of the study. Patients reported high satisfaction with brodalumab, with mean scores of 87.9 for efficacy, 83.0 for ease of use, and 88.8 for overall satisfaction. The highest PASI 100 response rates were observed in patients with moderate psoriasis at baseline. Brodalumab was effective in both biologic-naïve patients and those who had previously used other biologic treatments (anti-TNFα, anti-IL-17A, or IL-23/12 or IL-23 inhibitors) [73].

Tampouratzi et al. [74] showed that five patients initially responded very well to brodalumab, with significant improvements in the PASI and DLQI scores. The mean baseline PASI score was 18.58 and the median DLQI was 19.3. After a mean of 23 months, patients experienced secondary loss of efficacy of brodalumab treatment. Three patients developed or had worsening arthritic symptoms. Patients were then switched to adalimumab for a mean of 4.2 months. However, the change was not effective in treating psoriasis. Therefore, brodalumab treatment was resumed. Interestingly, resumption of brodalumab treatment resulted in the rapid remission of psoriasis, with PASI scores decreasing to a mean of 1.84 and DLQI scores to a median of 2.3. A second course of brodalumab treatment maintained efficacy for a mean of 8.8 months (at follow-up). Importantly, the arthritis symptoms in three affected patients also improved after resuming brodalumab treatment. The researchers speculated that a temporary switch to adalimumab may have somehow restored the effectiveness of brodalumab, possibly by affecting the complex balance of cytokines and immunogenicity associated with the disease. They emphasized that the mechanism of action of the brodalumab based on inhibition of the IL-17 receptor makes this biologic highly effective in the treatment of psoriasis compared to TNF-alpha inhibitors such as adalimumab. This study suggested that switching back to brodalumab after a short course of adalimumab treatment may be a viable treatment strategy for patients with severe psoriasis who experience secondary loss of efficacy to brodalumab treatment [74].

Gaudet et al. [75] demonstrated the effectiveness of brodalumab in the treatment of plaque psoriasis. The study found statistically significant improvements in disease severity and patient-reported outcomes. PASI scores improved from a median of 13.9 to 1.8. Similarly, psoriasis-affected BSA had improved from a median of 16.6% to 2.5%. DLQI scores improved also from a median of 16.2 to 2.9. A high percentage of patients continued brodalumab treatment (89.9% overall). Continuation rates were similar in patients who had just started using biologics and those who had previously used biologics. Notably, continuation rates were also high in patients whose most recent biologic therapy was an IL-17A inhibitor like secukinumab or ixekizumab. Interestingly, the percentage of patients who remained on brodalumab treatment over time was 82.0% at 6 months, 69.9% at 12 months and 63.4% at 18 months. In summary, the authors concluded that brodalumab is effective in a real-world setting, leading to significant improvements in psoriasis severity and quality of life [75].

Reich et al. [76] compared the effectiveness of brodalumab and guselkumab in patients with psoriasis who had failed prior treatment with ustekinumab (an IL-12/23 inhibitor). Brodalumab tended to achieve a higher PASI 100 score at week 16 (53.4%) compared with guselkumab (35.9%). However, this difference was not statistically significant (*p* = 0.069). The odds ratio was 2.05, suggesting that brodalumab was twice as likely to achieve PASI 100. Brodalumab appeared to have a more rapid onset of action than guselkumab, with separation of the time to PASI response curves beginning as early as week 2. Both treatments improved quality of life measures. Although brodalumab appeared to be more effective than guselkumab, particularly in terms of the rapidity of action, the study was underpowered by premature termination. Therefore, no definitive conclusions regarding superiority can be drawn [76].

Tokuyama et al. [77] presented a case report of a patient with worsening psoriasis vulgaris following mRNA-1273 COVID-19 vaccination. It was a 43-year-old Japanese man with an 8-year history of psoriasis vulgaris, along with comorbidities of dyslipidemia, hyperuricemia, and obesity. After six months of phototherapy, the patient began treatment with brodalumab, which improved his skin symptoms. Seven months after starting brodalumab, the patient received two doses of the mRNA-1273 COVID-19 vaccine, one month apart. One month after the second vaccine dose, his psoriasis symptoms worsened. Additional NB-UVB therapy did not improve the skin. Nine months later, treatment with bimekizumab led to a marked improvement. The authors highlight the importance of being aware of potential psoriasis exacerbations following COVID-19 vaccination, even in patients receiving highly effective biological therapies like brodalumab. They emphasize that psoriasis is often associated with metabolic syndrome, which can be a risk factor for a more severe course of COVID-19 and for which additional COVID-19 vaccinations are often recommended. Therefore, vigilance is necessary in these patients [77].

### 3.6. Blockage of IL-23

#### 3.6.1. Risankizumab

Risankizumab is a humanized IgG1 monoclonal antibody that selectively blocks the p19 subunit of interleukin-23 (IL-23), a key cytokine involved in immune-mediated inflammation in psoriasis. By binding IL-23 and preventing its interaction with the IL-23 receptor, risankizumab inhibits downstream signaling and reduces inflammation [78].

Estevinho et al. [79] presented a case report of a 35-year-old man with a history of both plaque psoriasis and human immunodeficiency virus (HIV) (controlled with highly active antiretroviral therapy—HAART). His psoriasis had worsened, covering 22% of his BSA and resulting in a PASI score of 21. He did not respond well to topical treatments or acitretin. HIV was well managed with an undetectable viral load and a CD4 cell count of 512 cells/μL. The standard dosing regimen of risankizumab was followed: 150 mg subcutaneous injections at weeks 0 and 4, and every 12 weeks thereafter. The patient achieved a PASI 100 response by week 8. Over a 56-week follow-up period, the patient maintained the PASI 100 response. Importantly, the risankizumab treatment did not negatively impact his HIV management. His viral load remained undetectable, and his CD4 cell count remained stable. No significant adverse events were reported [79].

Orsini et al. [80] described four cases of patients with well-controlled HIV who also had psoriasis. All patients were treated with risankizumab (150 mg at weeks 0 and 4, and then every 12 weeks). The first patient was a 45-year-old man who was diagnosed with HIV in 2011 and had a history of psoriasis since the age of 25. He was treated with a rilpivirine/tenofovir alafenamide/emtricitabine for HIV. In addition, he had severe psoriasis at baseline (PASI 22, DLQI 17). The patient achieved complete skin clearance (PASI 0) after 16 weeks. No adverse events were spotted. The second patient was a 32-year-old man who was diagnosed with HIV and psoriasis in 2020. He was treated with doravirine/tenofovir disoproxil fumarate/emtricitabine for HIV. His psoriatic skin worsened by a PASI 30, and he had complete skin clearance (PASI 0) after 16 weeks with risankizumab. The third patient was a 58-year-old man with a history of AIDS (Pneumocystis jirovecii pneumonia in 1999, Kaposi sarcoma in 2000). He was taking rilpivirine/emtricitabine/tenofovir alafenamide for HIV (CD4+ cell count 439 cells/mm^3^, undetectable viral load). He had severe psoriasis (PASI 20) before taking risankizumab and achieved PASI 1 after 16 weeks of therapy. The last patient was a 52-year-old man, diagnosed with HIV in 1996, with psoriasis onset in 2018 after a leg amputation. He was taking lamivudine/dolutegravir for HIV (undetectable viral load). He had severe psoriasis (PASI 10) and achieved complete skin clearance with risankizumab after 16 weeks. Overall, all patients showed positive responses to risankizumab, with significant improvement in PASI scores, including complete clearance in some cases. Treatment was well tolerated with no adverse events or viral reactivation reported [80].

Bardazzi et al. [81] studied the effectiveness of risankizumab in the treatment of leg psoriasis compared to psoriasis in other body areas. The primary objective of the study was to compare the PASI score for the legs (Leg-PASI) with the total PASI score. While both Leg-PASI and overall PASI showed statistically significant improvement from baseline at weeks 16 and 28, the Leg-PASI improved less than the overall PASI. Linear regression analysis confirmed that the Leg-PASI improved at a slower rate (less steep slope) than the overall PASI, indicating that leg psoriasis responds more slowly to risankizumab. The difference in response between leg psoriasis and other body areas was statistically significant in the initial weeks of treatment (up to week 16). However, this difference diminished and was no longer statistically significant at week 28. The study suggests that even though the legs may respond more slowly, further improvement is possible with risankizumab therapy, even after six months. The authors conclude that risankizumab is an effective treatment for leg psoriasis, but it should be emphasized that the legs may exhibit a slower response compared with other areas of the body [81].

Orsini et al. [82] demonstrated the use of risankizumab in very severe plaque psoriasis involving difficult-to-treat areas. The study included patients with very severe plaque psoriasis, involving areas that are typically difficult to treat. The mean PASI score decreased significantly from 35.1 ± 5.1 at baseline to 3.0 ± 4.3 at week 16 (*p* < 0.001). This indicates a rapid and substantial improvement in psoriasis severity. This improvement was maintained throughout the study period (up to week 104). PGA and DLQI scores also showed improvement over the course of the study. The study showed that risankizumab is an effective and safe treatment for patients with very severe psoriasis, even in difficult-to-treat areas [82].

The results of the study presented by Gargiullo et al. [83] include a large cohort of patients with psoriasis treated with risankizumab. Significant and continuous improvement in PASI scores was observed throughout the 3-year study period. Mean PASI scores decreased substantially from 15.73 (SD 7.65) at baseline to 2.27 (3.22) at week 16, 1.15 (2.17) at week 28, 0.76 (1.66) at week 52, 0.44 (1.22) at week 104 (2 years), and 0.25 (0.51) at week 156 (3 years). Over fifty-four percent of patients achieved PASI 90, and 35.34% achieved PASI 100 at week 16. Sustained efficacy was observed with increasing response rates over time: at week 52, 81.44% achieved PASI 90; at week 104, 88.99% achieved PASI 90; and at week 156, 99.07% achieved PASI 90. PASI 100 rates also increased: at week 52, 65.72%; at week 104, 73.73%’ and at week 156, 74.77%. Complete or almost complete clearance was achieved in 100% of patients with scalp psoriasis, palm/sole psoriasis, and genital psoriasis and in 95% of patients with nail psoriasis. Data also indicate that risankizumab is particularly effective in treating psoriasis in difficult-to-treat areas like the scalp, palms/soles, and genitals [83].

Hsieh et al. [84] conducted a retrospective study of 30 Chinese patients receiving 75 mg of risankizumab for up to 52 weeks. The study compared outcomes based on prior biologic treatment (biologic-naive vs. biologic-experienced) and body weight. Patients who had not previously received biologic treatment for psoriasis (biologic-naive) showed significantly better PASI 50/75/90/100 responses at week 52 compared to those who had prior biologic therapy. This difference was also observed throughout the study period using a statistical model (GEE—generalized estimated equation). Although there was no statistically significant difference in PASI responses between patients weighing 65 kg or less and those weighing more than 65 kg, there was a trend toward treatment failure in the higher body weight group after week 40. Patients who were biologic-naive and weighed 65 kg or less showed sustained PASI 50/75/90 responses from weeks 16, 28, and 40, respectively. This suggests that this subgroup might be particularly well suited for the 75 mg dose. While PASI 75 was higher in nondiabetic patients, other PASI scores did not differ significantly between diabetic and nondiabetic patients. The study suggests that 75 mg risankizumab may be an effective treatment option for certain patients with psoriasis, particularly those who are biologic-naive and weigh 65 kg or less [84].

Strober et al. [85] reported the long-term efficacy of risankizumab in patients with psoriasis after one year of treatment. Overall, after 12 months, a significant proportion of patients achieved clear skin (IGA 0) or clear/almost clear skin (IGA 0/1). Specifically, 55.4% achieved IGA 0, and 74.4% achieved IGA 0/1. Similar results were observed for PASI scores, with 55.8% achieving PASI 100 and 65.6% achieving PASI 90. As expected, patients who had not previously received biologic treatment (biologic-naive) responded even better than those who were biologic-experienced. Over sixty-six percent of patients achieved IGA 0, 87.2% achieved IGA 0/1, 66.2% achieved PASI 100, and 78.9% achieved PASI 90. In patients who had biological treatment, 45.4% of psoriatic patients achieved IGA 0, and 46.7% of psoriatic patients achieved PASI 100. The mean PASI score significantly improved from baseline to 12 months across all groups; overall, 88.1% improvement; biologic-naïve, 94.4% improvement; and biologic-experienced, 82.3% improvement. Beyond skin clearance, the study also reported improvements in DLQI and reductions in psoriasis symptoms like fatigue, skin pain, itch, and work/activity impairment [85].

Hamm et al. [86] described the effectiveness of switching to risankizumab in patients with moderate-to-severe plaque psoriasis who had not responded well to guselkumab. The authors focused on patients who had failed previous therapies, such as guselkumab, indicating a population with potentially more resistant psoriasis. Switching to risankizumab after guselkumab failure resulted in significant improvements in static PGA scores. Precisely, sPGA scores were lower at both 4 months and 12 months after starting risankizumab compared with those before switching. They found that nearly half of patients achieved an sPGA score of 0 or 1 after 4 months. This percentage increased substantially to 90% of patients achieving an sPGA score of 0 or 1 after 12 months. The authors concluded that risankizumab can be a valuable option for patients with moderate-to-severe plaque psoriasis who have not responded adequately to guselkumab [86].

Orsini et al. [87] presented results on the efficacy of risankizumab in the treatment of plaque psoriasis in difficult-to-treat areas. Risankizumab had high effectiveness across all difficult-to-treat areas: scalp: 97.58% of patients achieved a scalp-specific-PGA of 0 or 1 (clear or almost clear) at week 52; palms/soles: 95.28% achieved a palmoplantar-PGA of 0 or 1 at week 52; genitals: 100% achieved a static PGA-G (PGA of genitalia) of 0 or 1 at week 52; nails: 82% achieved a fingernail-PGA of clear or almost clear at week 52. A trend towards improvement in PGA scores from baseline to week 52 was observed in all domains, with the most significant improvements generally observed within the first 16 weeks and gradually maintained in subsequent weeks. The study provided strong evidence that risankizumab is effective and safe for treating moderate-to-severe plaque psoriasis in difficult-to-treat locations like the scalp, palms/soles, genitals, and nails [87].

#### 3.6.2. Guselkumab

Guselkumab is a fully human monoclonal antibody (IgG1λ) targeting the p19 subunit of IL-23, blocking its receptor binding and stopping the inflammatory cascade [88].

Marcelli et al. [89] showed the effectiveness and safety of guselkumab, a treatment that is likely to be used in psoriasis. The study identified a group of patients they call “super responders” (SRes) who had a particularly strong positive response to guselkumab. The main difference between SRes and non-super responders (nSRes) was the achievement and maintenance of PASI 100 (Psoriasis Area and Severity Index 100), which indicates the complete resolution of psoriasis symptoms. At week 204 (four years), 86.8% of SRes maintained PASI 100 compared to 62.8% of nSRes. At week 52, the difference was even greater: 92.5% of SRes maintained PASI 100 compared with only 31% of nSRes patients. Guselkumab significantly reduced the mean PASI score for the entire study population over time, from 13.9 at baseline to 0.8 at week 52, and remained at 0.8 up to week 204 [89].

Puig et al. [90] focused on long-term skin clearance in patients treated with guselkumab for psoriasis. More than half of the patients who achieved and maintained PASI 0 through 156 weeks did so by week 24 of guselkumab treatment. DLQI scores, which measure the impact of psoriasis on quality of life, improved alongside PASI scores. The mean DLQI score started at 13.5 and rapidly improved to 1.6 by week 16. At week 252, the vast majority of patients achieved a very good quality of life: 75.7% patients had a DLQI of 0 and 88.6% participants had a DLQI of 0 or 1 [91].

Megna et al. [91] investigated the long-term effectiveness (2 years) of guselkumab in psoriatic patients who had failed prior treatment with at least one anti-IL-17 medication (secukinumab, ixekizumab, brodalumab, or a combination). The average PASI was 12.8, and the average BSA was 24.5, indicating moderate-to-severe psoriasis at baseline. Guselkumab showed significant improvement in PASI scores over time. At week 16, 60.7% of patients achieved PASI 90, and 37.7% of patients achieved PASI 100. Furthermore, at week 104 (2 years), 73.8% people achieved PASI 900, and 59.0% participants achieved PASI 1000. Moreover, guselkumab was effective in treating psoriasis in difficult-to-treat areas (scalp, palms/soles, nails, genitals). Improvement in nails and palms/soles was initially slower but caught up by week 28 [91].

Soenen et al. [92] investigated the relationship between guselkumab dose (therapeutic concentration—TC) and clinical response in real-world psoriasis patients. The authors identified an optimal steady-state guselkumab TC of 1.6 μg/mL. Patients with concentrations at or above this level tended to have better responses. The median PASI score decreased significantly over time: at baseline, 7.0 (moderate psoriasis); at week 4, 3.1; at week 20, 0.6; at week 52, 0.2 [92].

Yuan et al. [93] investigated the role of IRF7 (Interferon Regulatory Factor 7) in psoriasis and how it is affected by guselkumab treatment. Guselkumab treatment led to the decreased expression (reduction in activity) of 799 genes. Analysis showed a strong enrichment of the IL-17 pathway. This is consistent with what we know about guselkumab action, as it targets IL-23, which is upstream of IL-17 in the inflammatory cascade of psoriasis. By reducing IL-23, guselkumab indirectly reduces IL-17 activity. The study found that the M2 module (likely a specific group of interacting genes or proteins) showed the greatest difference in activity. In addition, strong interactions were observed between keratinocytes (skin cells) and immune cells, which is important in psoriasis. The IRF7 regulon was found to be significantly involved in both psoriasis and treatment response. This was confirmed by Gene Set Enrichment Analysis using the IL-17 signaling pathway as a reference. Immunohistochemical analysis showed significant differences in IRF7 protein levels in psoriatic skin samples before and after 12 weeks of guselkumab treatment. This provides direct evidence that guselkumab affects IRF7 expression. The study suggests that the therapeutic effect of guselkumab in psoriasis may be mediated, at least in part, through its impact on IRF7 [93].

Strober et al. [94] monitored guselkumab treatment in a large group of patients with psoriasis and psoriatic arthritis over a long period of time. The analysis included 4399 guselkumab-treated patients, followed for a total of 10,787 patient-years. This large sample size provides robust safety data. Importantly, the adverse event rates in the guselkumab group remained stable throughout the long-term treatment period. No adverse effects of Crohn’s disease, ulcerative colitis, or active tuberculosis were reported in patients treated with guselkumab. In fact, after the placebo-controlled period, the rates of most adverse events were lower in the guselkumab group compared with the placebo-controlled period. This suggests that the long-term use of guselcumab does not increase the risk of adverse events [94].

Herranz-Pinto et al. [95] demonstrated the efficacy of guselkumab in the treatment of psoriasis. Guselkumab significantly reduced the PASI score to 2.1 between weeks 11 and 20, and this improvement was maintained throughout the 90-week study period. The drug survival rate was high at week 52. The study found that off-label dosing regimens (adjustments to the standard dosage) were as effective as the recommended dosage in the Summary of Product Characteristics (SmPC). The largest dose adjustments were made in patients who were either new to biologics (bio-naïve) or who were SR, with a 40% and 47% reduction in administrations, respectively, compared to the SmPC regimen. SR patients achieved an average PASI score of 0.1, compared to 3.4 in non-super responders (nSRs). All 100% of SR patients achieved PASI scores of ≤1, ≤3, and ≤5, compared to 39%, 70.7%, and 82.9% of nSR patients, respectively. Between weeks 71 and 90, SR patients maintained a very low average PASI score of 0.4, compared to 3.2 in nSR patients. The percentage of SR patients achieving PASI scores of ≤1, ≤3, and ≤5 was 90%, 90%, and 100%, respectively, compared to 38.5%, 65.4%, and 80.8% in nSR patients. In summary, the study demonstrates the effectiveness of guselkumab in treating psoriasis, with a particularly strong response observed in a subgroup of super responders, often those who were new to biologic treatments [95].

Jo et al. [96] compared the effectiveness of guselkumab, adalimumab, and placebo in the treatment of difficult-to-treat psoriasis areas. Guselkumab was significantly superior to adalimumab in achieving a “clear” or “near clear” scalp (85.7% vs. 67.3%, *p* = 0.004). Similarly, guselkumab showed a trend towards superiority over adalimumab for hands and/or feet psoriasis (82.9% vs. 61.5%, *p* = 0.054). On the opposite, guselkumab and adalimumab showed similar efficacy for fingernail psoriasis (63.6% vs. 54.8%, *p* = 0.412). Improvements in NAPSI scores were comparable between guselkumab and adalimumab (39.9% vs. 35.9%, *p* = 0.618). Guselkumab demonstrated greater clearance of scalp and hands/feet psoriasis compared to adalimumab, regardless of whether patients had previously used biological treatments (treatment-naïve or treatment-experienced) [96].

Zhdanava et al. [97] compared the long-term efficacy of guselkumab with two IL-17 inhibitors, secukinumab and ixekizumab, in patients with psoriasis. Guselkumab had significantly greater persistence than both secukinumab and ixekizumab. At 18 months, the persistence rate for guselkumab was approximately twice as high as that of secukinumab (HR = 2.15; *p* < 0.001) and ixekizumab (HR = 1.77; *p* < 0.001). Six months after discontinuation of the initial biologic, patients in the guselkumab group were significantly more likely to achieve remission compared with both the secukinumab (RR = 1.31; *p* < 0.001) and ixekizumab (RR = 1.40; *p* < 0.001) groups. After 6 months of follow-up, 43.7% of patients treated with guselkumab achieved remission compared with 31.1% of patients treated with ixekizumab. For partial remission, the numbers were 58.6% for guselkumab and 47.9% for ixekizumab. Guselkumab was 40% more likely to achieve remission and 22% more likely to achieve partial remission than ixekizumab (*p* < 0.001) [97].

Part et al. [98] supported the use of guselkumab in the treatment of generalized pustular psoriasis (GPP). The patient with GPP was treated with guselkumab, administered subcutaneously at a dose of 100 mg at weeks 0 and 4, followed by maintenance doses every 8 weeks. After 3 months of treatment, significant improvement was observed and the PGA score decreased from 3 to 0. This indicates a substantial resolution of pustular skin lesions. The DLQI score improved significantly from 26 to 4. Moreover, the Disease Activity Score 28-C-Reactive Protein (DAS28-CRP) score decreased from 3.39 to 2.17. This indicates a reduction in disease activity in the patient’s psoriatic arthritis, changing from moderate to low disease activity. Due to the positive response to guselkumab, the patient’s corticosteroid therapy was gradually reduced and completely discontinued within 4 weeks of starting guselkumab therapy. Importantly, no recurrence of GPP symptoms or new flares were observed after discontinuation of corticosteroids [98].

#### 3.6.3. Tildrakizumab

Tildrakizumab is a humanized IgG1κ monoclonal antibody that selectively binds the p19 subunit of interleukin-23 (IL-23), a key cytokine involved in the pathogenesis of psoriasis. By binding IL-23 and preventing it from interacting with the IL-23 receptor, tildrakizumab inhibits downstream inflammatory signaling and reduces inflammation in psoriasis [99].

Abu-Hilal et al. [100] presented a study on tildrakizumab for psoriasis. The study showed the progressive improvement in PASI scores over time, including the absolute scores and percentage improvements at weeks 16, 24, and 48. The PASI score was improved from 16.1 ± 6.7 at baseline to 3.1 ± 3.0 at week 16, 1.8 ± 2.0 at week 24, and 1.3 ± 1.7 at week 48. Additionally, 93% of the patients (40 out of the 43 patients who were assessed at week 48) achieved a PGA score of 0 or 1 [100].

Butron-Bris et al. [101] studied the association between genetic polymorphisms and tildrakizumab response. The researchers identified three genotypes associated with a lower probability of achieving PASI ≤ 1, for instance, GG for rs610604 (TNFAIP3), CTGT/- for rs72167053 (PDE4D) and CT for rs9373839 (ATG5). Additionally, they found one genotype associated with a higher probability of achieving PASI ≤ 1, CT for rs708567 (IL17RC). Moreover, the authors showed that 81% of patients achieved a PASI score of 3 or less at 6 months, and this increased to 87% at 12 months. A total of 76% of patients achieved a PASI score of 2 or less at 6 months, increasing to 81% at 12 months. A total of 63% of patients achieved a PASI score of 1 or less at 6 months, and this increased to 71% at 12 months. A total of 38% of patients achieved a PASI score of 0 (complete clearance) at 6 months, increasing to 42% at 12 months. The overall improvement in psoriasis severity with tildrakizumab treatment over time was highlighted [101].

Melgosa-Ramos et al. [102] evaluated the mid-term efficacy of tildrakizumab in real-world Spanish clinical practice. The authors reported the high overall tildrakizumab survival rate and the achievement of PASI ≤ 3 at weeks 28 and 52. A high proportion of patients showed significant improvements in PASI scores: 91.3% of patients achieved a PASI ≤ 3 by week 28; by week 52, 96.5% achieved a PASI ≤ 3. Additionally, 65.2% of patients reached a PASI = 0 (clear skin) by week 52. On the other side, the mean DLQI improved notably: at week 28, the mean DLQI was 1.3 (SD, 2.42), and by week 52, the mean DLQI dropped further to 0.83 (SD, 1.47). Summing up, the results from this study underscore tildrakizumab’s high efficacy [102].

Di Brizzi et al. [103] further supported the effectiveness of tildrakizumab in the treatment of moderate-to-severe plaque psoriasis. Firstly, the authors observed a significant reduction in the PASI score by week 12, decreasing from 19.2 ± 8.5 (at the baseline) to 3.5 ± 2.7 (*p* < 0.001). Subsequently, at week 48, the mean PASI score reduced to 0.6 ± 1.5 (*p* < 0.001). Secondly, a significant reduction in BSA was observed at week 12 (*p* < 0.001) with further improvement at week 48. Thirdly, the DLQI score also showed significant improvement, with a significant decrease at week 12 and a further decrease at week 48 (*p* < 0.001) [103].

Berenguer-Ruiz et al. [104] studied the efficacy and safety of tildrakizumab in a large cohort of patients with moderate-to-severe plaque psoriasis. The PASI reduced (from 10.7 to 1.7), and the BSA (decreased from 13.2 to 1.6) and DLQI changed (plummeted from 12.5 to 1.2), providing strong evidence of tildrakizumab’s effectiveness. The authors did not find that the correlation between effectiveness and factors like gender, obesity, psoriatic arthritis, and prior biologic treatment exposure strengthens the argument for tildrakizumab’s stable efficacy. The rate of adverse events was very low (5.9%). The most common adverse effect was infections (four out of eleven). In addition, one patient experienced a bleeding stroke and another patient died, but the cause of death was unrelated to the study [104].

Krygier et al. [105] presented a retrospective case series of the population (individuals with HIV and psoriasis) who were treated with tildrakizumab. The authors emphasized the significant clinical improvements (>95% improvement in PASI scores). The authors did not mention the adverse effects. A stable, undetectable HIV viral load was also observed during the study. To sum up, tildrakizumab proved to be a safe and effective treatment in this specific group of patients [105].

Burlando et al. [106] highlighted the long-term efficacy of tildrakizumab. The study evaluated PASI 100 at different time points (4 weeks, 16 weeks, and the range from 36 weeks to 3 years). Approximately, twenty-two percent of patients achieved PASI 100 by week 4 of therapy, and 51.2% gained PASI 100 by week 16. This enhancement was constant over the next 3 years, while the PASI remained at around 1.8 at 1 year, 2 years, and 3 years, indicating the efficacy and the minimal recurrence of psoriasis [106].

## 4. Psoriasis Management Challenges Regarding Difficult-to-Treat Areas

Traditional psoriasis severity metrics, such as the PASI and BSA, are widely acknowledged to underestimate the true disease burden, particularly when it involves anatomically complex or difficult-to-treat areas, including the scalp, nails, palmoplantar surfaces, and genital area. Although more specialized indices—such as the Psoriasis Scalp Severity Index (PSSI), NAPSI, and the Erythema, Scaling, Induration, and Fissuring (ESIF) scores—lead to a more clinical assessment of these sites, there are no present guidelines on how to effectively integrate these data into global PASI or DLQI frameworks. This diagnostic gap highlights the necessity of a nuanced, patient-centric approach to therapeutic decision-making. Specifically, the selection of biologic agents—particularly the high-potency IL-17 and IL-23 inhibitors—should be guided not only by total body surface area but also by the presence of these resistant areas. Recent evidence suggests that under-recognizing special area involvement often leads to clinical undertreatment and a profound impact on quality of life. Consequently, the presence of localized but high-impact disease may justify more ambitious therapeutic targets, such as PASI 90 or PASI 100, necessitating the early introduction of targeted biological therapies to achieve complete skin clearance and improved patient outcomes [107].

### 4.1. Biological Treatment of Psoriasis in Patients with Comorbidities

According to the EADV guidelines for the systemic treatment of psoriasis, the choice of biological therapy is heavily influenced by specific comorbidities to ensure optimal, safe treatment for both skin and related conditions. Psoriatic arthritis biological treatments include TNF inhibitors (adalimumab, certolizumab pegol, etanercept, infliximab), IL-17 agents (secukinumab, ixekizumab) and IL-12/23 inhibitors (ustekinumab). In the treatment of moderate-to-severe Crohn’s disease, while anti-TNF agents (infliximab, adalimumab) have traditionally been considered the primary, first-line biologic choice, IL-23 agents (guselkumab, tildrakizumab, risankizumab) have emerged as a powerful, often preferred, second-line option (particularly after anti-TNF failure) and, increasingly, a first-line option, especially for patients with specific comorbidities like psoriasis.

For ulcerative colitis, there is a similar regimen for some TNF inhibitors, with ustekinumab listed as a first-line drug and IL-23 agents as second-line drugs (guselkumab, tildrakizumab, risankizumab) and apremilast. Furthermore, in inflammatory bowel disease (IBD) TNF inhibitors are preffered, while IL-17 inhibitors should be avoided or used with caution, as they may exacerbate IBD. TNF inhibitors are contraindicated in advanced heart failure, whereas in ischemic heart disease, TNF inhibitors, IL-17, IL-12/23, and IL-23 agents are generally permitted. For patients with concomitant latent/treated tuberculosis, apremilast, IL-17, and IL-23 agents are preferred. During pregnancy, certolizumab is considered the preferred option due to minimal placental transfer [108]. In summary, although TNF inhibitors are still widely used as first-line agents, the therapeutic algorithm is changing. IL-23 inhibitors (specifically, risankizumab and guselkumab) are highly effective second-line treatment options and are increasingly used in the first-line setting, particularly given their superior and durable clinical remission.

### 4.2. Biologics Versus Traditional Systemic Therapies for Psoriasis

Biologics represent a significant advancement in the treatment of moderate-to-severe plaque psoriasis compared to conventional systemic therapies and phototherapy. Traditional systemic therapies, such as methotrexate, acitretin, and cyclosporine, are characterized by broad immunosuppressive effects or system-wide modulation of cell turnover. These agents are primarily administered orally, either daily or weekly. Their use necessitates frequent laboratory monitoring, including complete blood counts and metabolic panels, to screen for specific toxicities, e.g., methotrexate is associated with hepatotoxicity, cyclosporine with renal impairment, and acitretin with dyslipidemia. While these traditional medications are clinically effective, they generally have a slower onset of action, often taking weeks to months for maximal effect, with cyclosporine being the exception, as it acts rapidly to induce remission [109].

Importantly, biologics offer superior efficacy and faster response compared to traditional systemic drugs, which are generally used as first-line or in resource-limited settings despite the higher organ toxicity. Biological treatments for psoriasis, specifically TNF, IL-17, and IL-23 inhibitors, target key pro-inflammatory cytokines in the IL-23/IL-17 pathway to reduce inflammation. These agents, delivered via subcutaneous injection or intravenous infusion at intervals ranging from weeks to months, offer high rates of skin clearance [110].

Newer agents like IL-23 and IL-17 inhibitors (e.g., secukinumab, ixekizumab, brodalumab, guselkumab, risankizumab, tildrakizumab) offer greater specificity and potentially better overall safety than older TNF-α blockers (e.g., etanercept, infliximab, adalimumab), in particular, which show superior clearance for scalp, nail, and genital psoriasis (difficult areas). Mastorino et al. [111], in their large retrospective study, provided data that no significant efficacy alterations were spotted between IL-17 and IL-23 agents in terms of PASI 90/PASI 100 responses; however, IL-23 presented a higher early complete response (PASI < 3) in bio-naive patients after 16 weeks [111].

Biologics generally present a lower risk of cumulative organ toxicity compared to traditional systemics; however, the primary safety concern remains the risk of serious infections. Mandatory baseline monitoring includes screening for latent tuberculosis and hepatitis, followed by less frequent routine laboratory assessments than conventional therapies. In clinical trials, these agents consistently demonstrate superior efficacy, achieving higher skin clearance rates such as PASI 90 and PASI 100 [110].

### 4.3. Laser Therapy in Psoriasis

Laser-based phototherapy is a targeted treatment option primarily used in localized plaque psoriasis. The 308 nm excimer laser, which delivers high-intensity monochromatic UVB radiation to lesional skin, has been shown to induce T-cell apoptosis and reduce keratinocyte hyperproliferation. Clinical studies report that treatment administered two to three times weekly leads to significant improvement, with many patients achieving ≥75% reduction in local PASI scores after several sessions. Adverse effects are generally localized and include erythema, blistering, and transient hyperpigmentation [112,113,114]. The 595 nm pulsed dye laser (PDL), targeting dermal vasculature via selective photothermolysis, has demonstrated reductions in erythema, scaling, and plaque thickness in randomized controlled trials [115,116]. The 1064 nm Nd:YAG laser has also shown statistically significant reductions in modified PASI scores in small clinical studies [117]. In nail psoriasis, PDL treatment has been associated with improvements in NAPSI scores, although responses vary [118]. Moreover, the effectiveness of the Nd:YAG laser for hair removal has been shown in patients with hypertrichosis [119]. Systematic reviews emphasize the heterogeneity among studies and the need for larger randomized trials to define standardized protocols [114,120].

A summary of studies on biologic treatments for psoriasis is presented in Table 2.

### 4.4. Strengths and Limitations of Biological Treatment

Biological therapies have revolutionized the management of moderate-to-severe plaque psoriasis. These agents, particularly IL-17 (secukinumab, ixekizumab) and IL-23 inhibitors (risankizumab, guselkumab), demonstrate superior performance, with many patients achieving PASI 90 or higher. Biologicals, specifically IL-17 inhibitors, have shown a faster time to response (roughly 3–4 days in some studies) compared to conventional treatments (approx. 6 days). Effective, rapid skin clearance leads to improved patients’ quality of life. These treatments are also associated with lower risks of organ toxicity and can be effective for related conditions like psoriatic arthritis.

Limitations of biological treatment of psoriasis include high cost, potential loss of efficacy over time, and risks of infections or specific side effects.

## 5. Conclusions

To sum up, biological treatments have revolutionized psoriasis care, targeting specific immune pathways (like TNF-α, IL-17, IL-23) for significant skin clearance (PASI 90/100) and improved quality of life. Newer agents like IL-23 and IL-17 inhibitors (e.g., secukinumab, ixekizumab, brodalumab, guselkumab, risankizumab, tildrakizumab) offer greater specificity and potentially better overall safety than older TNF-α blockers (e.g., etanercept, infliximab, adalimumab), in particular, which show superior clearance for scalp, nail, and genital psoriasis. IL-23 and IL-17 inhibitors target key downstream inflammatory pathways more precisely, leading to fewer widespread immune suppressive effects and better outcomes. The so-far experience and rigorous supervision have shown that biologics can be safely administered with appropriate monitoring. However, all types of biologics may possess some adverse events, but the general safety profile is acceptable. Factors such as comorbidities, genetic markers, and lifestyle can predict positive or negative responses to biologic therapy. Undoubtedly, a personalized approach can not only improve the effectiveness of treatment but also reduce the risk of side effects, ultimately leading to better outcomes and patient satisfaction.

## Figures and Tables

**Table 1:** Examples and comparison of biologics.

Feature	TNF-α Inhibitors	IL-12/23 Inhibitors	IL-17 Inhibitors	IL-23 Inhibitors
**Examples**	Adalimumab, Etanercept, Infliximab	Ustekinumab	Secukinumab, Ixekizumab, Brodalumab, Bimekizumab	Guselkumab, Risankizumab, Tildrakizumab
**Primary Target**	Tumor necrosis factor-alpha	IL-12 and IL-23 (p40 subunit)	IL-17A (or IL-17 receptor/IL-17F)	IL-23 (p19 subunit)
**Efficacy (PASI\ 90/100)**	Moderate to high.	Moderate; slightly lower than IL-17/23 classes.	Very high: Rapid induction and high clearance.	The highest: Exceptional long-term skin clearance.
**Dosing Frequency**	Weekly to every 2 weeks (often).	Every 12 weeks (after loading).	Every 2 to 4 weeks (typically).	Every 8 to 12 weeks (maintenance).
**Possible adverse effects**	Risk of heart failure, demyelination, and tuberculosis reactivation.	Generally well tolerated; very few class-specific risks.	Risk of mucocutaneous candidiasis (thrush) and inflammatory bowel diseases and flares.	Low risk of serious infection; generally fewer adverse events.

**Table 2:** Summary of the studies on biologic treatment of psoriasis.

Type of Biologic Treatment	Study Design	Model	Key Observation	Reference
			**Blockage of T cells**	
Alefacept	Multicenter (51 centers in the USA and Canada), randomized, double-blind, parallel-group study comparing	553 patients with psoriasis	First and second courses of alefacept were generally effective and well tolerated	Krueger et al. [22]
			**Blockage of TNF-alpha**	
Adalimumab	Delphi consensus study	36 patients with psoriasis	Adalimumab is a potentially effective and cost-effective first-line treatment option	Matucci-Cerinic et al. [26]
Adalimumab	Prospective observational study	33 patients with psoriasis	Adalimumab therapy was associated with improvements in sperm motility, vitality, and testosterone levels	Yuksek et al. [27]
Adalimumab	Prospective cohort study	6575 patients with psoriasis	Patients on adalimumab were significantly more likely to achieve PASI ≤ 2 compared to those on methotrexate	Alabas et al. [28]
Adalimumab	Multicenter, prospective, observational, daily practice cohort study	135 patients with psoriasis	Patients who achieved PASI 75 had significantly higher adalimumab trough levels (6.07 mg/L) compared to those who did not reach this level of improvement (2.99 mg/L)	Menting et al. [29]
Etanercept	Peer-reviewed original research article presenting real-world evidence from a retrospective observational study	43 patients with psoriasis	A high percentage of patients achieved substantial improvement in their psoriasis symptoms within 16 weeks of etanercept treatment	Narbutt et al. [31]
Etanercept	Peer-reviewed original research article in the form of a case report	1 patient with psoriasis	Within 2 weeks of etanercept treatment, the patient experienced nearly total remission of skin lesions	Lin et al. [32]
Etanercept	Peer-reviewed original research article in the form of a case report	1 patient with psoriasis	The patient was diagnosed with paradoxical colitis likely triggered by etanercept	Huynh et al. [33]
Certolizumab	Peer-reviewed original research article presenting real-world evidence from a prospective, multicenter, non-interventional observational study	399 patients with psoriasis	Precisely, high rates of PASI 75 (77% of patients) and PASI 90 (56.5% of patients) responses were observed at 12 months, indicating significant skin clearance	Korge et al. [35]
Certolizumab	Retrospective observational study	66 patients with psoriasis	The mean absolute PASI score decreased by 8 points (−78.4%), indicating a substantial improvement in psoriasis severity	Ruiz et al. [36]
Certolizumab	Computational modeling study	patients with psoriasis	The model of vPop predicted clinical outcomes (like PASI scores) and molecular markers of psoriasis severity	Coto-Segura et al. [37]
			**Blockage of IL-12/IL-23**	
Ustekinumab	Randomized, double-blind, Phase III clinical trial	249 patients with psoriasis	SB17, a biosimilar to UST, was clinically comparable to UST up to week 28 in patients with moderate-to-severe plaque psoriasis	Feldman et al. [39]
Ustekinumab	Real-world observational study	98 patients with psoriasis	Patients without metabolic syndrome had a significantly higher rate of achieving PASI 75, and, what is more, lower triglyceride levels were associated with a higher likelihood of achieving PASI 75	Li et al. [40]
Ustekinumab	Retrospective observational cohort study	160 patients with psoriasis	A high percentage (80.55%) of non-naïve patients responded positively to ustekinumab treatment	Gönülal et al. [41]
Ustekinumab	Case report, literature review	1 patient with psoriasis	A 71-year-old man with psoriasis who developed bullous pemphigoid (BP) twice after receiving ustekinumab injections	Kong et al. [42]
Ustekinumab	Case report, literature review	1 patient with psoriasis	39-year-old woman with Crohn’s disease who developed a psoriasiform rash while on ustekinumab treatment	Olteanu et al. [43]
			**Blockage of IL-17**	
Ixekizumab	Multicenter retrospective study	120 patients with psoriasis	High efficacy was observed due to ixekizumab intake. In the scalp, 96% of patients achieved clear or almost clear skin after a year	Valenti et al. [45]
Ixekizumab	Retrospective observational study	108 patients with psoriasis	Not only did ixekizumab achieve significant improvements in PASI 75 responses, but also in PASI 90 and 100 responses, respectively	Burlando et al. [46]
Ixekizumab	Retrospective observational study	43 patients with psoriasis	Ixekizumab appears to have a more substantial impact on the metabolome compared to adalimumab in patients with psoriasis	Deng et al. [47]
Ixekizumab	Retrospective observational study	128 patients with psoriasis	The authors found that MHR significantly decreased after treatment with ixekizumab	Tamer et al. [48]
Ixekizumab	Case report	1 patient with psoriasis	Ixekizumab postpartum led to the complete resolution of the pustules and erythema in a patient with GPPP	Ozdemier et al. [49]
Ixekizumab	Pooled analysis of clinical trial data from two Phase III randomized controlled trials	736 patients with psoriasis	Ixekizumab performed significantly better than etanercept, regardless of prior biologic exposure	Gottlieb et al. [50]
Ixekizumab	Observational, non-interventional retrospective study	255 patients with psoriasis	Ixekizumab offers superior long-term efficacy and tolerability compared to secukinumab for psoriasis treatment, as evidenced by longer drug survival and lower switching rates	Bucur et al. [51]
Ixekizumab	Retrospective observational study	306 patients with psoriasis	Ixekizumab had the highest PASI 100 achievement rate (71.2%) among other biologics	Ting et al. [52]
Secukinumab	Prospective observational study	676 patients with psoriasis	In short, the study found that the standard dose of secukinumab was highly effective and well tolerated	Papp et al. [54]
Secukinumab	Retrospective observational multicenter cohort study	347 patients with psoriasis	SEC-DR was successful in 77.8% of the patients, meaning they maintained their PASI response until the end of the study	Dauden et al. [55]
Secukinumab	Real-world clinical practice data	173 patients with psoriasis	Several SNPs that may help predict response to secukinumab	Munoz-Aceituno et al. [57]
Secukinumab	Comparative cohort design	11 patients with psoriasis	The authors found significant changes in the gut microbiota of patients after secukinumab treatment	Du et al. [57]
Secukinumab	Retrospective observational real-world clinical data	123 patients with psoriasis	Insufficient response was the most common reason for stopping secucinumab treatment	Tada et al. [58]
Secukinumab	Case report	1 patient with psoriasis	The patients treated with secukinumab for one year developed an eczematoid lesion	Ma et al. [59]
Secukinumab	Case report	1 patient with psoriasis	The patient experienced a new onset of AA after the initiation of secukinumab	Choi et al. [60]
Secukinumab	Case report	1 patient with psoriasis	An eczematous-like reaction potentially linked to secukinumab	Zundell et al. [61]
Bimekizumab	Case report	1 patient with psoriasis	PASI 90 was observed within 72 h and PASI 100 within one week after bimekizumab treatment	Abdin et al. [63]
Bimekizumab	Retrospective observational real-world clinical cohort study	65 patients with psoriasis	Bimekizumab demonstrated high effectiveness in treating plaque psoriasis	Rompoti et al. [64]
Bimekizumab	Prospective observational real-world clinical study	56 patients with psoriasis	Interestingly, at baseline, the biologic-naive group had significantly higher PASI and DLQI scores (more severe disease and worse quality of life) than the biologic-experienced group	Megna et al. [65]
Bimekizumab	Single-center retrospective observational study	19 patients with psoriasis	Younger patients might have a better initial response to bimekizumab	Hagino et al. [66]
Bimekizumab	Pooled analysis of Phase 2/3 randomized controlled clinical trials	2480 patients with psoriasis	Screening and monitoring questionnaires also indicated low levels of SIB and depression among patients receiving bimekizumab	Blauvelt et al. [67]
Bimekizumab	Multicenter, randomized, double-blind, active comparator- and placebo-controlled Phase III clinical trial	183 patients with psoriasis	The study concluded that bimekizumab is more effective than both ustekinumab and placebo for treating moderate-to-severe plaque psoriasis	Reich et al. [68]
Bimekizumab	Phase III randomized, double-blind, placebo-controlled clinical trial	349 patients with psoriasis	The study showed that these high response rates were maintained through to week 56 with both the every 4-week and every 8-week bimekizumab dosing schedules	Gordon et al. [69]
			**Blockage of IL-17RA**	
Brodalumab	Prospective multicenter non-interventional observational study	638 patients with psoriasis	Brodalumab was effective across different patient subgroups in patients with psoriasis	von Kiedrowski et al. [71]
Brodalumab	Phase IV multicenter open-label clinical study	251 patients with psoriasis	Brodalumab can be an effective treatment option for psoriasis patients who have not responded adequately to other biologic therapies	Papp et al. [72]
Brodalumab	Prospective multicenter observational study (real-world, non-interventional, non-controlled)	44 patients with psoriasis	High satisfaction with brodalumab, with average scores of 87.9 for efficacy, 83.0 for ease of use, and 88.8 for overall satisfaction	Gkalpakiotis et al. [73]
Brodalumab	Observational study	6 patients with psoriasis	Switching back to brodalumab after a short course of adalimumab might be a viable strategy for managing severe psoriasis patients who experience secondary loss of efficacy with brodalumab	Tampouratzi et al. [74]
Brodalumab	Retrospective observational cohort study (real-world data analysis)	864 patients with psoriasis	Brodalumab is effective in real-world settings, leading to significant improvements in psoriasis severity and quality of life	Gaudet et al. [75]
Brodalumab	Phase IV randomized, multicenter, double-blind clinical trial with active comparator	113 patients with psoriasis	Brodalumab showed a tendency towards a higher rate of PASI 100 at week 16 (53.4%) compared to guselkumab (35.9%)	Reich et al. [76]
Brodalumab	Case report	1 patient with psoriasis	The authors highlighted the importance of being aware of potential psoriasis exacerbations following COVID-19 vaccination, even in patients receiving highly effective biological therapies like brodalumab	Tokuyama et al. [77]
			**Blockage of IL-23**	
Risankizumab	Case series (report of two individual patient cases)	1 patient with psoriasis and HIV infection	The risankizumab treatment did not negatively impact HIV management	Estevinho et al. [79]
Risankizumab	Case series (multiple individual patient clinical observations).	4 patients with psoriasis	Overall, all patients with HIV infection showed positive responses to risankizumab, with significant improvement in PASI scores, including complete clearance in some cases	Orsini et al. [80]
Risankizumab	Retrospective observational cohort study (real-world clinical data analysis)	124 patients with psoriasis	Leg-PASI improved at a slower rate (less steep slope) than the overall PASI, indicating that leg psoriasis responds more slowly to risankizumab	Bardazzi et al. [81]
Risankizumab	Retrospective observational real-world clinical study (retrospective cohort)	27 patients with psoriasis	The study concluded that risankizumab is an effective and safe treatment for patients with very severe psoriasis, even when difficult-to-treat areas are involved	Orsini et al. [82]
Risankizumab	Retrospective multicenter observational cohort study (real-world evidence)	1047 patients with psoriasis	Risankizumab is particularly effective in clearing psoriasis in difficult-to-treat areas like the scalp, palms/soles, and genitals	Gargiullo et al. [83]
Risankizumab	Retrospective observational real-world clinical study (retrospective cohort)	30 patients with psoriasis	75 mg risankizumab may be an effective treatment option for certain patients with psoriasis, particularly those who are biologic-naive and weigh 65 kg or less	Hsieh et al. [84]
Risankizumab	Retrospective observational study using real-world registry data	287 patients with psoriasis	The mean PASI score significantly improved, due to risankizumab intake, from baseline to 12 months across all groups: overall, 88.1% improvement; biologic-naïve, 94.4% improvement; and biologic-experienced, 82.3% improvement	Strober et al. [85]
Risankizumab	Retrospective observational study	13 patients with psoriasis	The study expressed that risankizumab can be a valuable option for patients with moderate-to-severe plaque psoriasis who have not responded adequately to guselkumab	Hamm et al. [86]
Risankizumab	Retrospective observational cohort study (real-world data)	202 patients with psoriasis	The study provided strong evidence that risankizumab is effective and safe for treating moderate-to-severe plaque psoriasis in difficult-to-treat locations like the scalp, palms/soles, genitals, and nails	Orsini et al. [87]
Guselcumab	Retrospective observational real-world cohort study	232 patients with psoriasis	The study identified a group of patients they call “super responders” (SRes) who experience a particularly strong positive reaction to guselkumab	Marcelli et al. [89]
Guselcumab	Post hoc analysis of data from a Phase III randomized controlled clinical trial	329 patients with psoriasis	Long-term skin clearance in patients treated with guselkumab for psoriasis was observed	Puig et al. [90]
Guselcumab	Retrospective observational real-world clinical study	62 patients with psoriasis	Guselkumab was effective in treating psoriasis in difficult-to-treat areas (scalp, palms/soles, nails, genitals)	Megna et al. [91]
Guselcumab	Prospective multicenter observational cohort study	75 patients with psoriasis	The authors identified an optimal steady-state guselkumab TC of 1.6 μg/mL	Soenen et al. [92]
Guselcumab	Translational molecular research with observational components	3 patients with psoriasis	Immunohistochemical analysis showed significant differences in IRF7 protein levels in psoriatic skin samples before and after 12 weeks of guselkumab treatment	Yuan et al. [93]
Guselcumab	Integrated analysis (pooled analysis) of multiple Phase II/III randomized controlled trials	4399 patients with psoriasis	Long-term use of guselcumab does not increase the risk of adverse events	Strober et al. [94]
Guselcumab	Retrospective observational real-world clinical study	69 patients with psoriasis	Effectiveness of guselkumab in treating psoriasis, with a particularly strong response observed in a subgroup of super responders, often those who were new to biologic treatments	Herranz-Pinto et al. [95]
Guselcumab	Post hoc analysis of Phase III randomised controlled trial data	837 patients with psoriasis	Guselkumab demonstrated greater clearance of scalp and hands/feet psoriasis compared to adalimumab, regardless of whether patients had previously used biological treatments (treatment-naïve or treatment-experienced)	Jo et al. [96]
Guselcumab	Retrospective observational comparative cohort study (real-world data)	18,061 patients with psoriasis	Guselkumab demonstrated significantly greater persistence than both secukinumab and ixekizumab	Zhdanava et al. [97]
Guselcumab	Case report	1 patient with psoriasis	Guselkumab is an effective way of treating GPP	Part et al. [98]
Tildrakizumab	Multicenter retrospective observational (real-world) study	75 patients with psoriasis	Tildrakizumab is a great choice for the treatment of plaque psoriasis	Abu-Hilal et al. [100]
Tildrakizumab	Retrospective observational pharmacogenetic association study (real-world clinical data)	90 patients with psoriasis	It was described as a link between genetic polymorphisms and tildrakizumab response	Buron-Bris et al. [101]
Tildrakizumab	Retrospective multicenter observational real-world study	91 patients with psoriasis	The authors reported the high overall tildrakizumab survival rate and the achievement of PASI ≤3 at weeks 28 and 52	Melgosa Ramos et al. [102]
Tildrakizumab	Retrospective multicenter observational real-world study	51 patients with psoriasis	The researchers spotted the effectiveness of tildrakizumab in treating moderate-to-severe plaque psoriasis	Di Brizzi et al. [103]
Tildrakizumab	Retrospective observational multicenter cohort study (real-world data)	190 patients with psoriasis	The authors mentioned the lack of correlation between effectiveness and factors like gender, obesity, psoriatic arthritis, and prior biologic treatment exposure, which strengthened the argument for tildrakizumab’s consistent efficacy	Berenguer-Ruiz et al. [104]
Tildrakizumab	*Retrospective case series* (clinical case series)	3 patients with psoriasis and HIV infection	Tildrakizumab was shown to be a safe and efficacious modality in patients with psoriasis and HIV infection	Krygier et al. [105]
Tildrakizumab	Retrospective multicenter observational real-world study	136 patients with psoriasis	Tildrakizumab had a long efficacy of action and minimal recurrence of psoriasis	Burlando et al. [106], Strober et al. [94]

## Data Availability

No new data were created or analyzed in this study. Data sharing is not applicable to this article.

## Abbreviations

PASI—Psoriasis Area and Severity Index, ROC—receiver-operator characteristic, AUC—area under the curve, BSA—body surface area, CDLQI—Children’s Dermatology Life Quality Index, CZP—certolizumab pegol, PGA—Physician’s Global Assessment, NAPSI—Nail Psoriasis Severity Index, vPop—virtual population approach, PBPK—physiologically based pharmacokinetic, SB—Systems Biology, BP—bullous pemphigoid, MHR—monocyte-to-high-density lipoprotein cholesterol ratio, GPPP—generalized pustular psoriasis of pregnancy, IGA—Investigator’s Global Assessment, SNPs—single nucleotide polymorphisms, AA—alopecia areata, SIB—suicidal ideation and behavior, HAART—highly active antiretroviral therapy, Leg-PASI—PASI score for the legs, GEE—generalized estimated equation, SRe—super responder, nSRe—non-super responder, TC—therapeutic concentration, IRF7—Interferon Regulatory Factor 7, SmPC—Summary of Product Characteristics, GPP—generalized pustular psoriasis, DAS28-CRP—Disease Activity Score 28-C-Reactive Protein.
